# Biomechanical analysis of plate versus K-wire fixation for metacarpal shaft fractures with wedge-shaped bone defects

**DOI:** 10.1186/s12891-024-07482-2

**Published:** 2024-05-03

**Authors:** Yung-Cheng Chiu, Tsung-Yu Ho, Cheng-En Hsu, Chen-Wei Yeh, Yen-Nien Ting, Ming-Tzu Tsai, Jui-Ting Hsu

**Affiliations:** 1 School of Medicine, China Medical University, Taichung, 404 Taiwan; 2 Department of Orthopedic Surgery, China Medical University Hospital, Taichung, 404 Taiwan; 3 Department of Orthopaedics, Taichung Veterans General Hospital, Taichung, 407 Taiwan; 4 Sports Recreation and Health Management Continuing Studies-Bachelor’s Degree Completion Program, Tunghai University, Taichung, 407 Taiwan; 5 3D Printing Medical Research Center, China Medical University Hospital, Taichung, 404 Taiwan; 6 Department of Biomedical Engineering, Hungkuang University, Taichung, 433 Taiwan; 7 Department of Biomedical Engineering, China Medical University, Taichung, 404 Taiwan; 8 School of Dentistry, China Medical University, Taichung, 404 Taiwan

**Keywords:** Metacarpal shaft fracture, Wedge-shaped defect, Locked plate, K-wire

## Abstract

**Background:**

Metacarpal shaft fracture is a common type of hand fracture. Numerous studies have explored fixing transverse fractures in the midshaft of the metacarpal bone. However, this section of the metacarpal bone is often susceptible to high-energy injury, resulting in comminuted fracture or bone loss. In such cases, wedge-shaped bone defects can develop in the metacarpal shaft, increasing the difficulty of performing fracture fixation. Notably, the research on this type of fracture fixation is limited. This study compared the abilities of four fixation methods to fix metacarpal shaft fractures with wedge-shaped bone defects.

**Methods:**

In total, 28 artificial metacarpal bones were used. To create wedge-shaped bone defects, an electric saw was used to create metacarpal shaft fractures at the midshaft of each bone. The artificial metacarpal bones were then divided into four groups for fixation. The bones in the first group were fixed with a dorsal locked plate (DP group), those in the second group were fixed with a volar locked plate (VP group), and those in the third group were fixed by combining dorsal and volar locked plates (DP + VP group), and those in the fourth group were fixed with two K-wires (2 K group). Cantilever bending tests were conducted using a material testing machine to measure yielding force and stiffness. The four groups’ fixation capabilities were then assessed through analysis of variance and Tukey’s test.

**Results:**

The DP + VP group (164.1±44.0 N) achieved a significantly higher yielding force relative to the 2 K group (50.7 ± 8.9 N); the DP group (13.6 ± 3.0 N) and VP group (12.3 ± 1.0 N) did not differ significantly in terms of yielding force, with both achieving lower yielding forces relative to the DP + VP group and 2 K group. The DP + VP group (19.8±6.3 N/mm) achieved the highest level of stiffness, and the other three groups did not differ significantly in terms of stiffness (2 K group, 5.4 ± 1.1 N/mm; DP group, 4.0 ± 0.9 N/mm; VP group, 3.9 ± 1.9 N/mm).

**Conclusions:**

The fixation method involving the combined use of dorsal and volar locked plates (DP + VP group) resulted in optimal outcomes with respect to fixing metacarpal shaft fractures with volar wedge bone defects.

## Introduction

Metacarpal bones are a primary skeletal component of the palm, and the metacarpal shaft is crucial for hand prehension. Metacarpal fractures are common, accounting for approximately 40% of hand fractures [1], and such fractures can mostly be treated nonsurgically, with treatment methods such as splint immobilization often yielding satisfactory outcomes. However, surgical intervention may be required for metacarpal fractures that exhibit more complexity or unstable fracture patterns, especially oblique or spiral fractures or those causing metacarpal shortening [2–5]. Common clinical fixation methods include lag screw fixation, bone plate fixation, and K-wire fixation. However, no consensus has been reached regarding the appropriate fixation method for treating complex cases of metacarpal shaft fractures, such as comminuted fractures or those involving bone loss [3, 6]. K-wire fixation is a minimally invasive surgical technique that violates least blood circulation and soft tissue at the fracture site, thereby enhancing the bone union. However, for comminuted metacarpal shaft fractures or cases involving wedge-shaped bone defect, whether K-wire fixation can provide sufficient fixation strength at the fracture site remains unclear [7]. Generally, lag screw fixation is excluded as a treatment option for comminuted metacarpal shaft fractures or cases involving bone loss because this surgical procedure is challenging and that may cause fractured bone shortening. The disadvantages of bone plate fixation include the requirement for extensive soft tissue dissection, which may lead to poor preservation of blood circulation at the fracture site [8–10]. Although bone plate fixation provides greater fracture fixation strength relative to other methods, no consensus has been reached regarding the optimal position and number of plates for the procedure. Therefore, by conducting a biomechanical study, we identified the optimal fixation method for treating comminuted metacarpal shaft fractures or those involving bone loss.

When metacarpal shaft fractures occur because of falling or punching, the most common resulting fracture patterns are transverse, oblique, or spiral fractures. These types of fracture generally exhibit good cortical bone contact after fracture reduction, allowing for effective bone healing when the appropriate fixation strength is applied [5, 11]. However, in highly complex situations (e.g., fractures caused by crushing injuries, the recalcitrant nonunion of fractures, or the development of osteomyelitis), bone absorption is likely to occur at the site of a metacarpal shaft fracture. In such cases, good cortical bone contact after fracture reduction is unlikely to achieve because of partial bone loss. Consequently, the fracture healing process is prolonged, and greater fracture fixation strength is required to support bone union.

Plate fixation is commonly used to treat metacarpal shaft fractures, and most hand surgeons have agreed that this method provides a stronger fixation strength in comparison to other fixation methods. However, the optimal placement of bone plates on the metacarpal shaft remains unclear. Notably, how the strongest fixation strength can be achieved when bone loss occurs at the site of a metacarpal shaft fracture remains a subject of debate. Most metacarpal shaft fractures involving bone loss occur on the volar side because the intrinsic muscles of the hand generate a traction force on the fracture’s end, causing dorsal angulation deformity [12, 13]. Consequently, the volar side cortical bone of the fracture end experiences compression force [14]. If proper fracture fixation is not performed during the acute stage, the volar cortical bone may eventually experience bone absorption resulting in bone loss at the fracture site [15].

Numerous studies have explored methods for fixing horizontal or oblique fractures at the metacarpal shaft. However, no study has comprehensively discussed methods for fixing a fracture in the midshaft of a metacarpal bone with wedge-shaped bone defects. In the present study, we used artificial metacarpal bones that simulated the material properties of real cortical bone and cancellous bone to compare the abilities of four methods—fixation with a volar locked plate, fixation with a dorsal locked plate, fixation involving the combined use of dorsal and volar locked plates, and fixation with two K-wires—to fix a fracture in the midshaft of a metacarpal bone with wedge-shaped bone defects.

## Materials and methods

### Preparation of artificial metacarpal bone specimens

In this study, artificial third metacarpal bones (Sawbones, Vashon, WA, USA) were employed because of the considerable challenge of acquiring a sufficient number real human metacarpal bones with comparable bone quality and size. In total, 28 artificial metacarpal bone specimens were used in the present study. Due to the joint capsule and muscle attachments at both the metacarpal head and metacarpal base, fractures often occur at the more fragile metacarpal shaft, especially under injury mechanisms that create a torsion force, resulting in a comminuted metacarpal shaft fracture. This often causes a wedge-shaped bone defect on the volar side. We created a uniform wedge-shaped bone defect fracture model in the middle of the metacarpal shaft to investigate which fixation method would achieve the best mechanical strength. This facilitates a more reference-worthy biomechanical study on fracture fixation methods [16–18].

### Fixation methods

All specimens were assigned to undergo one of four fixation techniques performed by a single senior hand surgeon (Yung-Cheng Chiu). An electric saw created a wedge-shaped bone defect on the volar side of all the artificial bones. The wedge-shaped bone defect had a base-side measuring 0.6 cm and a perpendicular height of 1.0 cm.

**Fig. 1:**
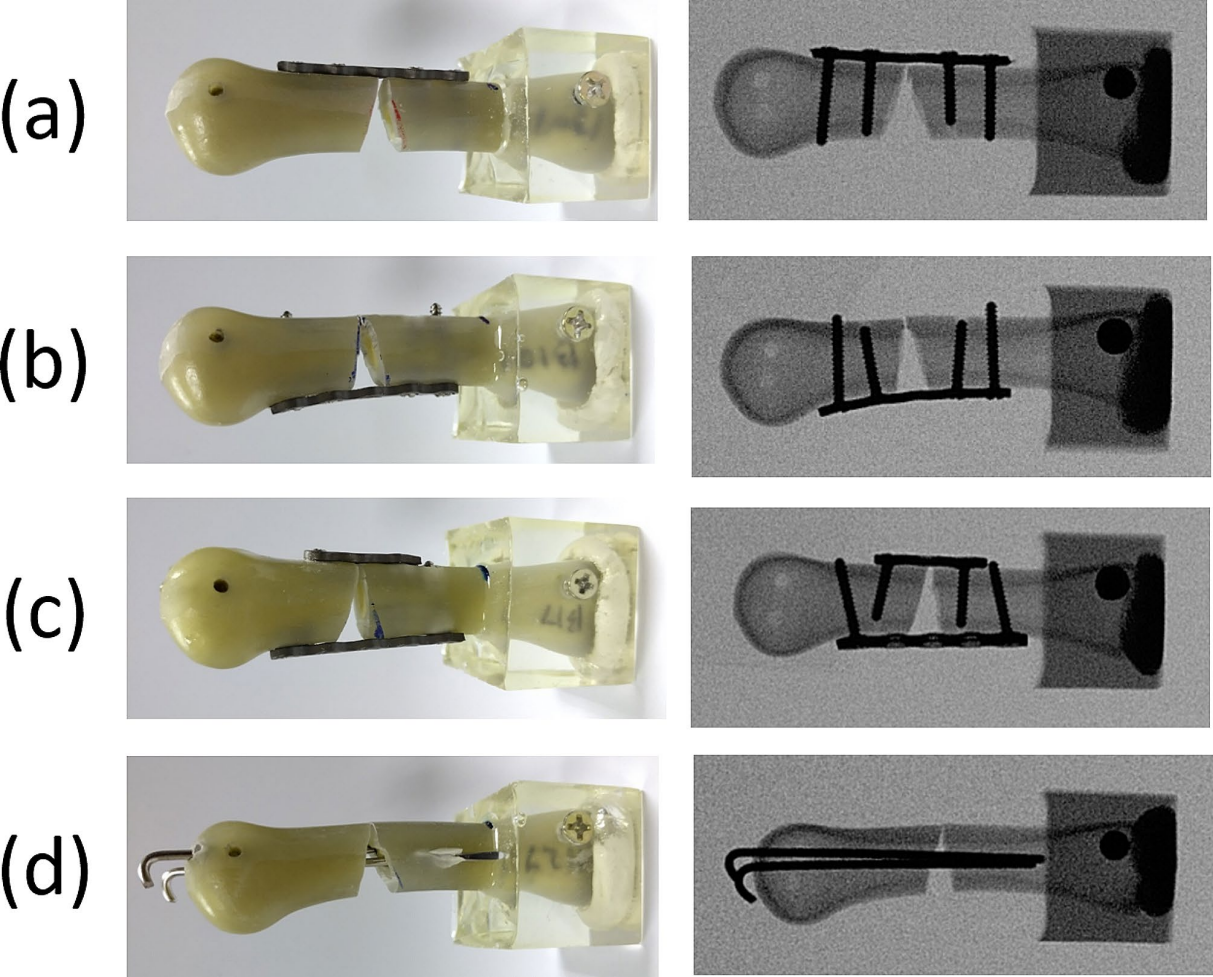
Photographs (left) and radiographs (right) of four types of fixation methods involving the use of (**a**) a locked plate with four locked bicortical screws on the dorsal side, (**b**) a locked plate with four locked bicortical screws on the volar side, (**c**) a combination of a 3-hole locked plate with two locked bicortical screws on the dorsal side and a 5-hole locked plate with two locked bicortical screws on the volar side, and (**d**) two K-wires in a cross-pin fixation configuration

- Group 1: Seven specimens were assigned to the dorsal locked plate (DP) group and stabilized using a 5-hole straight locked plate that was secured with four locked screws with a 2.3-mm diameter (Stryker, Freiburg, Germany). Initially, the 5-hole locked plate was positioned on the dorsal side of the metacarpal shaft and centered on the fracture site. Subsequently, two bicortical locked screws were inserted distally to the fracture site, after which two bicortical locked screws were inserted proximally to the fracture site. Throughout the surgical procedure, fracture alignment was maintained through manual axial compression (Fig. 1a).
- Group 2: Seven specimens were assigned to the volar locked plate (VP) group and stabilized using a 5-hole straight locked plate that was secured with four locked screws with a 2.3-mm diameter (Stryker, Freiburg, Germany). Initially, the 5-hole locked plate was positioned on the volar side of the metacarpal shaft and centered on the fracture site. Subsequently, two bicortical locked screws were inserted distally to the fracture site, after which two bicortical locked screws were inserted proximally to the fracture site. Throughout the surgical procedure, fracture alignment was maintained through manual axial compression (Fig. 1b).
- Group 3: Seven specimens were assigned to the dorsal and volar locked plate (DP + VP) group. These specimens were stabilized using one 5-hole straight locked plate and one 3-hole straight locked plate that were secured with four locked screws with a 2.3-mm diameter (Stryker, Freiburg, Germany). Initially, the 5-hole locked plate was positioned on the volar side of the metacarpal shaft and centered on the fracture site. Subsequently, one bicortical locked screws were inserted distally to the fracture site, after which one bicortical locked screws were inserted proximally to the fracture site. The 3-hole locked plate was then positioned on the dorsal side of the metacarpal shaft and centered on the fracture site. One bicortical locked screw was then inserted distally to the fracture site, after which one bicortical locked screw was inserted proximally to the fracture site. Throughout the surgical procedure, fracture alignment was maintained through manual axial compression (Fig. 1c).
- Group 4: Seven specimens were assigned to the two K-wire (2 K) group and secured using two K-wires (diameter, 1.4 mm) that were inserted distally from the metacarpal head; these K-wires traversed through the intramedullary canal of the fracture site and emerged proximally by punching out from the lateral side cortex in the supracondylar region. The two K-wires were applied in a cross-pin fixation configuration, and fracture reduction was maintained through manual axial compression throughout the surgical procedure (Fig. 1d).

### Biomechanical test

**Fig. 2:**
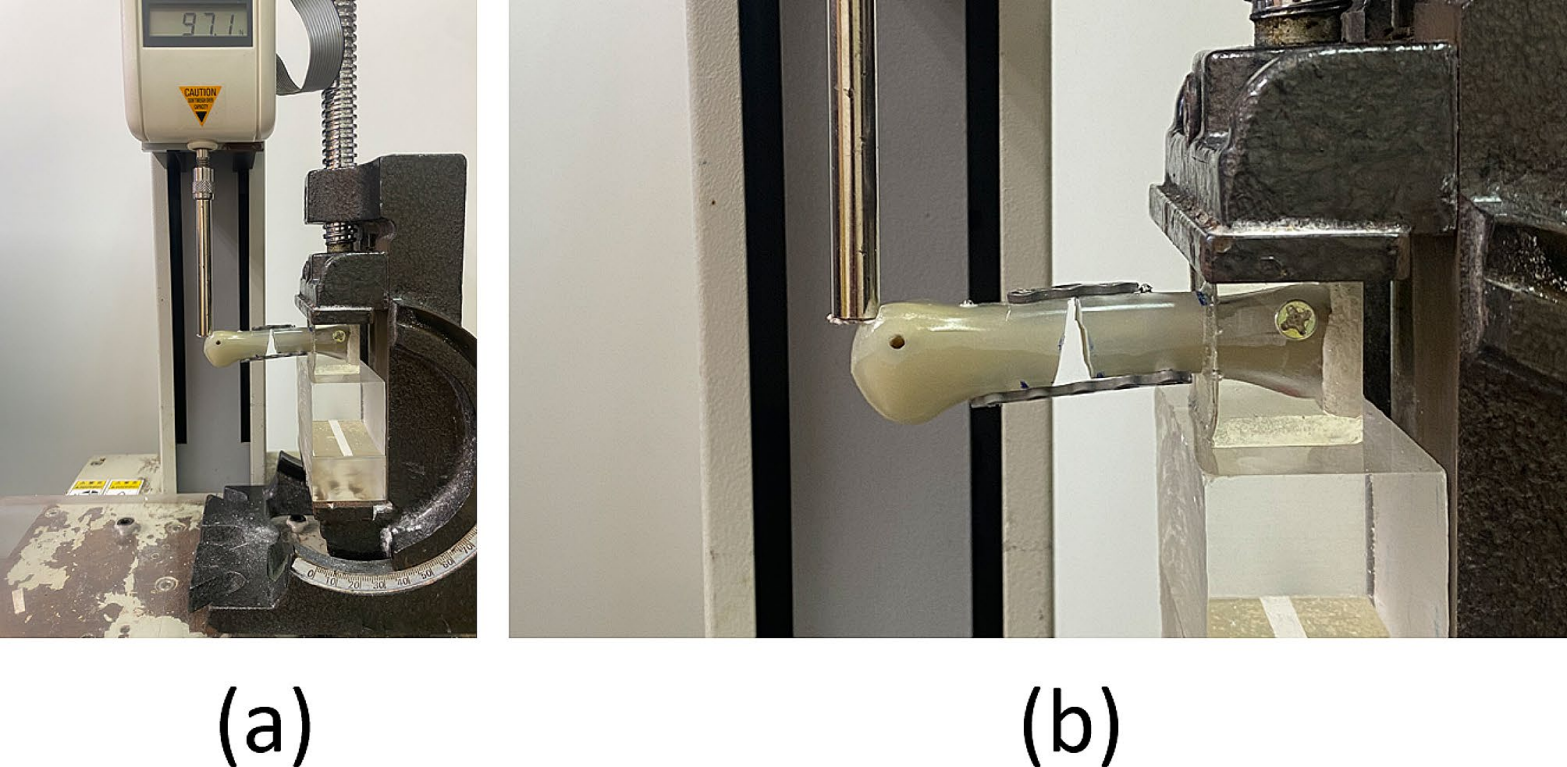
Experimental setup: (**a**) full view and (**b**) closeup view

Based on the research protocols of other studies [19–22], the present study performed cantilever bending tests to evaluate the four tested methods. Prior to conducting cantilever bending tests, we used molded epoxy clamps to securely hold the proximal end of each artificial third metacarpal bone in a custom fixture. The tests were performed using a material testing system (JSV-H1000, Japan Instrumentation System, Nara, Japan; Fig. 2). A perpendicular load was applied to the dorsal side of a specimen at a point 50 mm distant from the fixture until the specimen failed. In each test, a loading speed of 10 mm/min was maintained. Force-displacement data were recorded, enabling us to determine the maximum fracture force experienced by each tested specimen and its stiffness.

### Statistical analysis

The present study summarized the results (presented as means and standard deviations) pertaining to the maximum fracture force and stiffness of the specimens with metacarpal shaft fractures, which were treated using five fixation methods. First, one-way analysis of variance and Tukey’s test were conducted at a significance level of 0.05 to compare the fracture force and stiffness of the specimens with fractures. All statistical analyses were performed using SPSS Version 19 (IBM Corporation, Armonk, NY, USA).

## Results

**Fig. 3:**
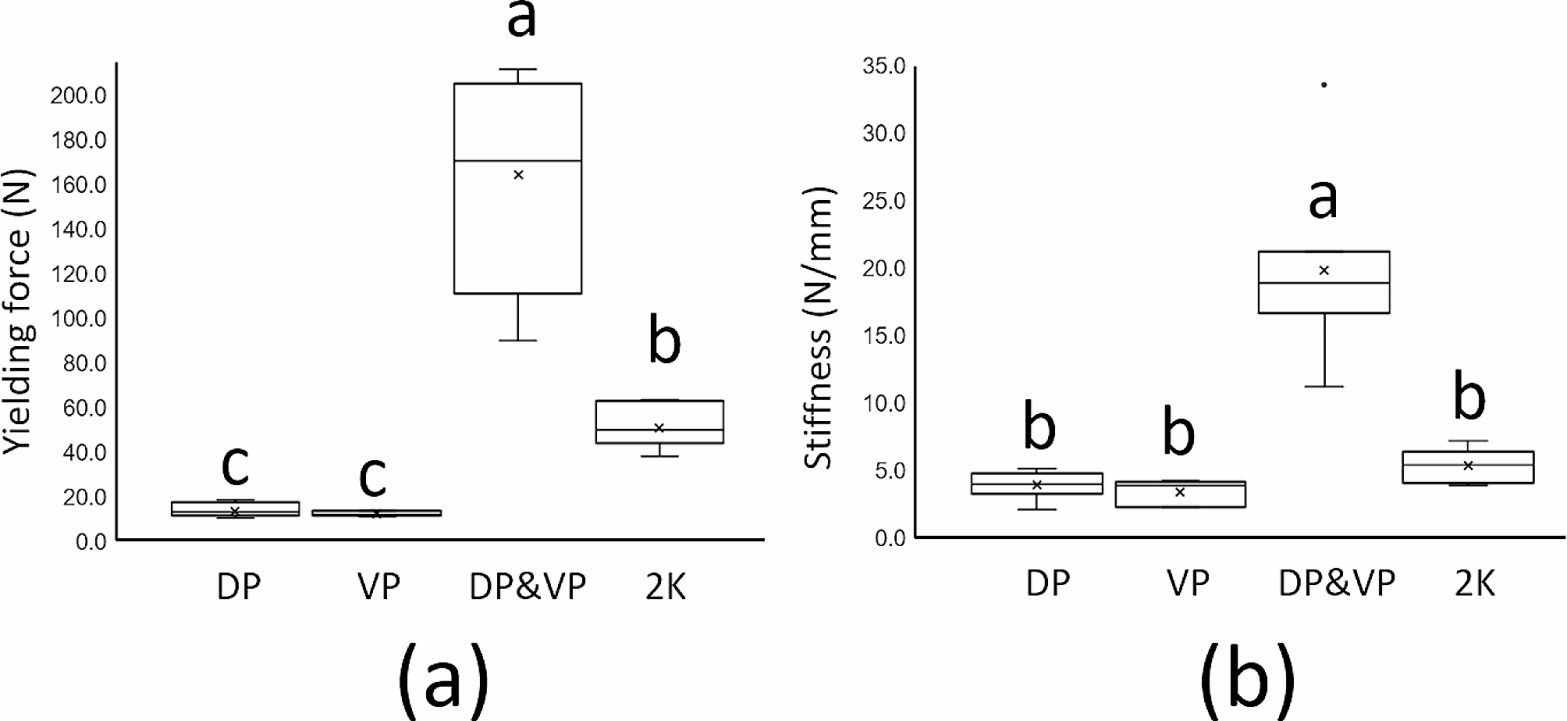
Box plot presenting yielding force (**a**) and stiffness (**b**) achieved through the four fixation methods. Post hoc pairwise comparisons were conducted using Tukey’s test. In this box plot, identical lowercase English letters within groups indicate non-statistically significant differences at the 0.05 significance level. Groups: DP group, group in which specimens underwent fixation involving the use of a locked plate with four locked bicortical screws on the dorsal side; VP group, group in which specimens underwent fixation involving the use of a locked plate with four locked bicortical screws on the lateral side; DP + VP group, group in which specimens underwent fixation involving the combined use of a locked plate with two locked bicortical screws on the dorsal side and a locked plate with two locked bicortical screws on the lateral side; 2 K group, group in which specimens underwent fixation involving the use of two K-wires

The experimental results for yielding force and stiffness are presented in Table 1. Regarding yielding force (Fig. 3a), the DP + VP group (mean ± standard deviation, 164.1 ± 44.0 N) achieved a significantly higher yielding force value relative to the 2 K group (50.7 ± 8.85 N), and the DP group (13.6 ± 3.0 N) and VP group (12.3 ± 1.0 N) achieved lower levels of yielding force relative to the other four groups but did not differ significantly from each other (*P* > 0.05). For stiffness (Fig. 3b), the DP + VP group (19.8 ± 6.3 N/mm) achieved the highest level of stiffness, and the other three groups did not differ significantly in terms of stiffness (*P* > 0.05).

**Table 1:** Yielding force (N) and stiffness (N/mm) achieved through four fixation methods

		Group	Sample size	Mean	SD	CV	MAX	MIN	*P*†
Yielding force (N)		DP	7	13.6	3.0	21.7	18.7	10.4	< 0.001
		VP	7	12.3	1.0	8.6	13.8	11.0	
		DP&VP	7	164.1	44.0	26.8	211.5	89.9	
		2 K	7	50.7	8.8	17.3	63.4	37.9	
Stiffness (N/mm)		DP	7	4.0	0.9	23.7	5.2	2.1	< 0.001
		VP	7	3.9	1.9	24.3	4.3	2.3	
		DP&VP	7	19.8	6.3	31.9	33.6	11.2	
		2 K	7	5.4	1.1	20.5	7.2	3.9	

Groups: DP group, group in which specimens underwent fixation involving the use of a locked plate with four locked bicortical screws on the dorsal side; VP group, group in which specimens underwent fixation involving the use of a locked plate with four locked bicortical screws on the lateral side; DP + VP group, group in which specimens underwent fixation involving the combined use of a locked plate with two locked bicortical screws on the dorsal side and a locked plate with two locked bicortical screws on the lateral side; 2 K group, group in which specimens underwent fixation involving the use of two K-wires.

† indicates one-way analysis of variance.

## Discussion

Metacarpal shaft fractures are among the most commonly reported hand fractures. Although most studies have focused on investigating and discussing methods for fixing transverse fractures at the midshaft of the metacarpal bone, clinical observations have indicated that wedge-shaped bone defects also frequently occur at this location [15]. Despite this, research on fixation methods specifically tailored for treating metacarpal shaft fractures accompanied by wedge-shaped bone defects remains scarce. The present study preliminarily used artificial bones to investigate the simultaneous use of locked plates on the volar and dorsal sides to treat midshaft metacarpal fractures with wedge-shaped defects, and it revealed that this method led to considerably more favorable fixation outcomes compared to methods that solely involve the use of a dorsal locked plate, a volar locked plate, or K-wires.

**Fig. 4:**
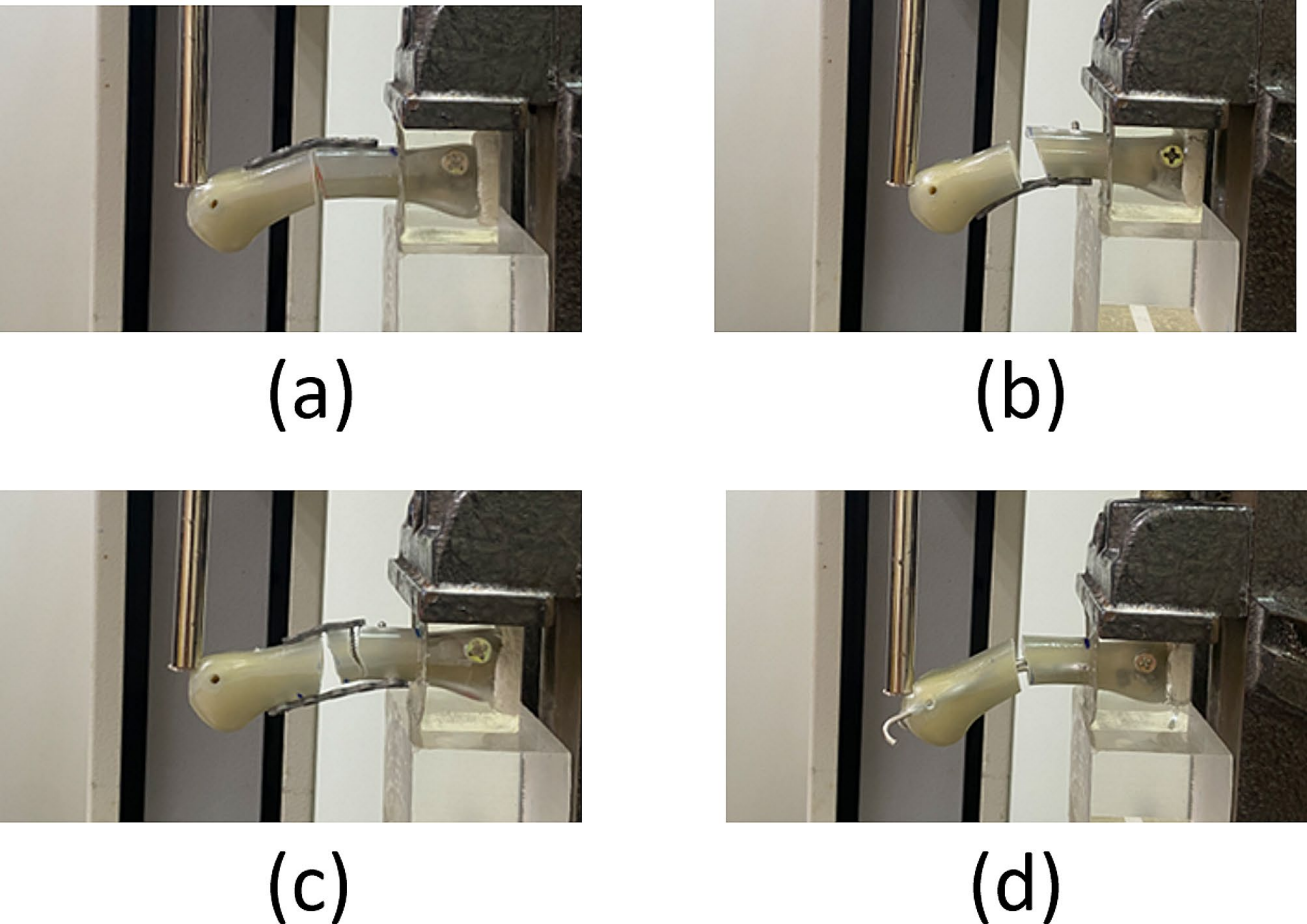
Photographs of specimens from four fixation groups after cantilever bending testing: (**a**) DP specimen, (**b**) VP specimen, (**c**) DP + VP specimen, and (**d**) 2 K specimen. Groups: DP group, group in which specimens underwent fixation involving the use of a locked plate with four locked bicortical screws on the dorsal side; VP group, group in which specimens underwent fixation involving the use of a locked plate with four locked bicortical screws on the volar side; DP + VP group, group in which specimens underwent fixation involving the combined use of a 3-hole locked plate with two locked bicortical screws on the dorsal side and a 5-hole locked plate with two locked bicortical screws on the volar side; 2 K group, group in which specimens underwent fixation involving the use of two K-wires

In this study, artificial metacarpal bones were utilized due to the challenges associated with obtaining cadaver bones. Another contributing factor is the prevalence of metacarpal fractures among young individuals, whose bodies, characterized by solid bone density, are less readily available for research. Even if accessible, obtaining a sufficient number of metacarpal bones from young cadavers presents logistical difficulties. Previous research has commonly employed either porcine metacarpal bones [23–25] or artificial alternatives [19, 27]. Our decision to utilize artificial bones aimed to ensure uniformity and consistency across all test samples, facilitating a fair comparison of fixation abilities among various osteosynthetic techniques. The selection of artificial bones aligns with guidelines provided by the American Society for Testing and Materials (F-1839-08) (17, 27–28), which recognizes artificial bones as the most appropriate material for such research endeavors. This choice also ensures compliance with ethical considerations regarding using human or animal specimens. We chose to conduct cantilever bending tests in this study, which, although slightly different from the physiological loading tests commonly employed in clinical settings, serve as a valuable method for evaluating fixation capabilities. It’s important to note that no existing in vitro biomechanical test perfectly replicates the exact physiological loading conditions. In addition to cantilever bending tests, previous research has utilized various other mechanical testing modes, such as the three-point bending test [26], modified three-point bending test [23, 24], four-point bending test [27], and torsional test [19]. Our study employs cantilever bending tests because relevant literature commonly utilizes this method [19, 21, 22]. Therefore, the present study conducted cantilever bending tests to evaluate the four tested methods. Because our experiment involved the simulation of metacarpal shaft fractures with wedge-shaped bone defects, the locked plates used in DP or VP fixation were likely to permanently deform without causing fractures when a cantilever bending test was performed (Fig. 4.). Therefore, the present study used yielding force as an evaluation criterion instead of maximum fracture force.

Numerous studies have identified the use of two K-wires as a standard method for fixing metacarpal shaft transverse fractures [2, 14]. Through this method, satisfactory results can be achieved because no bone loss occurs at the fracture site, and cortical bone contact is excellent after fracture reduction and fixation. Consequently, when patients begin grasping/prehension rehabilitation, K-wire fixation can create a tension band effect that allows for effective bone union on both the volar and dorsal side of cortex at the fracture site [28, 29]. On the other hand, when bone loss occurs on the volar side at metacarpal shaft fracture site, a patient who begins grasping/prehension rehabilitation experiences considerable stress at the fracture end on the volar side because of the lack of proper cortical bone contact. In such cases, the entire fixation may fail if the K-wire fixation cannot withstand the forces generated by the movement at the fracture end.

There is a notable scarcity of biomechanical studies comparing fixation methods in metacarpal shaft fractures with wedge-shaped bone defects. Gajendran et al. [30] conducted a biomechanical evaluation comparing the efficacy of double-row locking plates with single- and double-row non-locking plates using a comminuted metacarpal fracture model. This model involved creating a 3-mm gap in fourth-generation biomechanical testing grade composite sawbones by removing a 3-mm block of bone from the diaphysis at the midpoint of each metacarpal [30]. Wedge-shaped bone defects can manifest in any part of the bone, with the formation of the wedge fragment on either the volar or dorsal side, depending on the force causing the fracture. The defect’s size and shape significantly influence the stability of internal fixation. In clinical settings, the force-deforming metacarpal shaft fractures are typically directed towards the volar side, resulting in most malunited metacarpal fractures being dorsally angulated [16–18]. Our wedge-shaped bone defect metacarpal fracture model is designed to replicate clinical observations, showing that volar defects are more common than dorsal defects or a combination of both. Through our experiment, we demonstrated that the forces supported by K-wire fixation are comparable to those supported by dorsal or volar locked plate fixation. That is, when bone loss occurs on the volar side at the site of a metacarpal shaft fracture, single-plate fixation does not provide a mechanical advantage over two K-wires fixation. This insufficiency led to the K-wire breakage and permanent plate deformity. For the fixation module involving a combination of dorsal and volar locked plates, fracture occurred at the junction of the plate and bone, and no deformity occurred at the fracture site. These findings indicate that when bone defects occur on the volar side of a metacarpal shaft fracture, to enable patients to begin grasping/prehension training earlier, a combination of dorsal and volar locked plates should be recommended to be applied for fracture fixation. When fixation with K-wire fixation, single dorsal plate fixation, and single volar plate fixation, patients are advised to wear a plaster cast for an extended period until callus formation is visible on X-ray images. At this point, they can commence their rehabilitation program.

Similar to most other studies [19, 22, 31, 32], the present study conducted in vitro mechanical experiments by using artificial bones, which are commonly used in the fields of orthopedics and dentistry; this is because obtaining fresh human bones with similar strengths is difficult. Currently, no optimal design has been established for an in vitro mechanical experiment that simulates the forces applied to metacarpal bones experiencing volar wedge bone defects in the midshaft area. On the basis of the research protocols of other studies [19, 21, 22], the present study performed cantilever bending testing to assess the abilities of several fracture fixation methods. However, our study analyzed only four methods for fixing metacarpal shaft fractures with wedge-shaped bone defects. That is, it did not explore fixation methods such as intramedullary screw fixation, K-pin, and external fixation. Future studies should address this limitation by analyzing additional fixation methods to conduct a more comprehensive experimental analysis.

## Conclusion

The present study used artificial bones in a mechanical experiment involving cantilever bending tests. When a midshaft metacarpal fracture is accompanied by a wedge-shaped bone defect, a fixation method involving a combination of dorsal and volar locked plates can lead to more favorable fixation outcomes relative to methods involving a dorsal locked plate, a volar locked plate, or K-wires. Patients treated using a fixation method involving a combination of dorsal and volar locked plates are more likely to achieve successful fracture union and more favorable clinical functional outcomes.

## Data Availability

All data generated or analyzed during this study are included in this published article.
